# Elucidation of Novel Molecular Targets for Therapeutic Strategies in Urothelial Carcinoma: A Literature Review

**DOI:** 10.3389/fonc.2021.705294

**Published:** 2021-08-05

**Authors:** Blessie Elizabeth Nelson, Angelina Hong, Bagi Jana

**Affiliations:** ^1^Department of Hematology and Oncology, University of Texas Medical Branch, Galveston, TX, United States; ^2^School of Medicine, University of Texas Medical Branch, Galveston, TX, United States; ^3^Department of Hematology and Oncology, MD Anderson Cancer Center, Houston, TX, United States

**Keywords:** urothelial carcinoma, molecular targets, FDA approvals, bladder cancer, clinical trials, targeted therapy

## Abstract

Urothelial carcinoma therapy is a rapidly evolving and expanding field. Traditional cytotoxic chemotherapy regimens have not produced optimal long-term outcomes, and many urothelial cancer patients have comorbidities that disqualify them as chemotherapy candidates. In recent years, a plethora of novel therapeutic agents that target diverse molecular pathways has emerged as alternative treatment modalities for not only metastatic urothelial carcinoma, but also for muscle-invasive bladder cancer and non-muscle invasive bladder cancer in adjuvant and definitive settings. This review paper aims to discuss the various categories of therapeutic agents for these different types of urothelial cancer, discussing immunotherapy, antibody-drug conjugates, kinase inhibitors, CAR-T cell therapy, peptide vaccination, and other drugs targeting pathways such as angiogenesis, DNA synthesis, mTOR/PI3K/AKT, and EGFR/HER-2.

## Introduction

Bladder cancer or urothelial carcinoma (UC) of the bladder is a common and deadly malignancy worldwide, and chemotherapy has produced limited improvement in outcomes. There are 550,000 new cases of UC globally each year, and UC accounts for about 2.1% of all deaths due to cancer. Women have a 0.27% lifetime risk of acquiring UC, whereas this risk is 1.1% in men (1). Management of UC depends on whether the malignancy is invasive or non-invasive. Low to intermediate risk non-muscle invasive bladder cancer (NMIBC) is treated with transurethral resection of bladder tumor (TURBT) followed by surveillance. For high risk NMIBC, cystectomy or intravesical induction therapy with chemotherapy followed by Bacille Calmette-Guerin (BCG) maintenance therapy or BCG therapy with maintenance therapy may also be considered. For NMIBC patients refractory to BCG therapy, cystectomy is first-line management. Localized muscle-invasive bladder cancer (MIBC) is treated with radical cystectomy and neoajuvant chemotherapy or radical cystectomy in chemotherapy ineligible candidates or bladder preservation strategies with concurrent chemoirradiation. Upfront therapy for muscle-invasive UC is neoadjuvant chemotherapy in patients who are eligible to receive platinum-based therapy. Standard chemotherapy for metastatic UC is platinum-based, and includes three different drug regimens: methotrexate, vinblastine, doxorubicin, and cisplatin (MVAC), dose dense MVAC, and lastly gemcitabine with cisplatin (GC) (2). There has been extensive research on chemotherapy for UC; platinum-based therapy was discovered to improve outcomes in locally advanced UC patients in the 1980s, with MVAC being the first regimen studied (3). In 2000, a phase III trial comparing the outcomes in advanced/metastatic UC patients treated with MVAC versus GC found that the survival rates are similar in both arms, but GC therapy had fewer toxic effects that MVAC. Although the GC arm experienced more grade ¾ anemia and thrombocytopenia events, but the MVAC arm had a greater occurrence of grade ¾ neutropenia, neutropenic fever, neutropenic sepsis, mucositis, and alopecia (4). There are also alternative chemotherapy regimens for patients who are ineligible for cisplatin therapy. De Santis et al.’s 2012 phase II/III trial compared the outcomes of gemcitabine with carboplatin versus methotrexate with carboplatin and vinblastine (MCAVI) in patients unfit for cisplatin-based chemotherapy. They found that there were no significant differences in efficacy between the two arms, although the incidence of severe acute toxicities (e.g. death, grade 3 or 4 renal toxicity, neutropenic fever) occurred more frequently in the MCAVI group (5).

However, metastatic UC still carries a particularly poor prognosis. After treatment with these chemotherapy regimens, the approximate median survival is only thirteen to fifteen months (4, 6). There are a handful of possible molecular mechanisms that may explain this suboptimal response to cytotoxic chemotherapy. Recent studies have revealed that UC patient differences in the expression of various proteins, such as matrix metalloproteinase-7 and syndecan-1, affect the prognosis following chemotherapy (7, 8). Additionally, individual variations in tumor-infiltrating B-cell activity have significant prognostic value for MIBC patients (9), and long noncoding mRNAs have also been found to regulate muscle-invasive UC proliferation and resistance to chemotherapy (10). Considering this suboptimal response to chemotherapy, a growing body of research is exploring targeted molecular therapy as an alternative to traditional chemotherapy. Additionally, promising biomarkers such as tumor mutational burden continue to be investigated and can provide prognostic information to guide developing bladder cancer treatments such as immunotherapy (11).

The Cancer Genome Atlas (TCGA) is a practice changing cancer genomics program which molecularly characterized over 20,000 primary cancers and matched normal samples spanning 33 cancer types (12). In 2020, a molecular classification of MIBC on basis of 1750 MIBC transcriptomic profiles from 18 datasets comparing six molecular classification schemes was reported. Six molecular classes were identified namely luminal papillary, luminal non-specified, luminal unstable, stroma-rich, basal/squamous, and neuroendocrine-like (13). Each consensus class is associated with differing stromal and genetic alteration that could identify who would more likely respond to immunotherapy or targeted therapy (14).

This paper discusses these categories of novel therapy that each have diverse molecular targets, which can affect host immune response as well as tumor activity. There is a real unmet need for development of therapeutic strategies for BCG refractory NMIBC setting, perioperative setting in MIBC, and platinum unfit population. Literature for these various UC therapies will be reviewed in the context of localized and metastatic MIBC and NMIBC management. Firstly, the agents for UC management that are currently or formerly FDA-approved or have promising phase III trial results will be discussed. The UC therapies with only phase I or II trials or ongoing phase III trials will then be outlined. Although more research is still needed, the rapidly developing field of genomic sequencing and biomarker stratification holds promise for targeted molecular therapy in UC realm (15).

## Therapeutic Agents for Advanced or Metastatic Urothelial Carcinoma

### Immunotherapy

**Figure 1:**
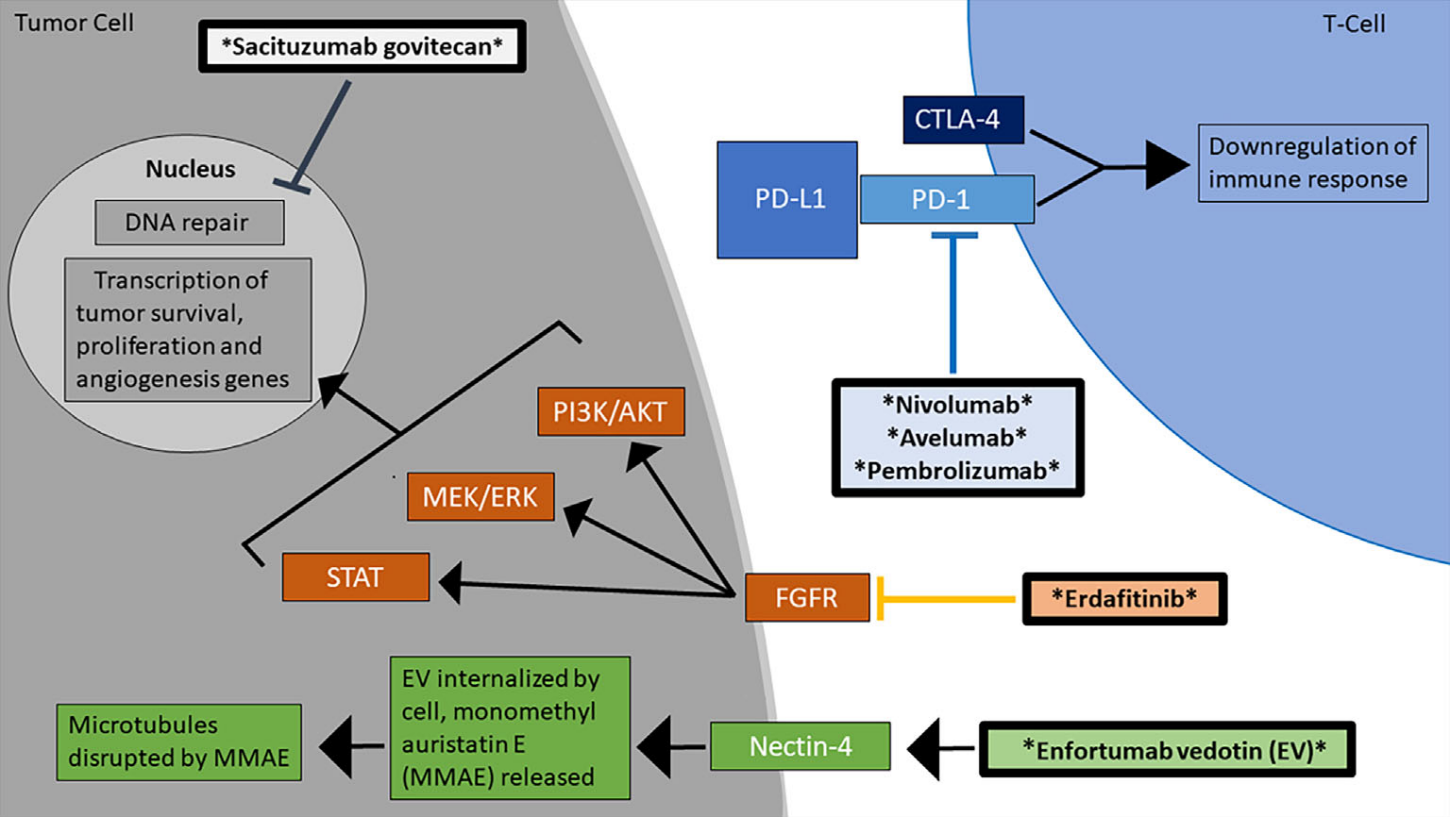
Mechanisms of action for the FDA-approved treatments for advanced/metastatic urothelial carcinoma. There are currently 6 FDA-approved agents for the treatment of MIBC, which work through diverse mechanisms, including checkpoint inhibition and blockage of the FGFR signaling pathway (16). The therapeutic agents are marked by asterisks and are in bolded boxes.

Checkpoint inhibitors comprise one of the most promising fields for metastatic or advanced UC therapy. There are two main categories of checkpoint inhibitor therapy: agents targeting programmed cell death protein 1 (PD-1) or programmed cell death-ligand 1 (PD-L1) and agents targeting cytotoxic T-lymphocyte-associated protein 4 (CTLA-4) in **Figure 1**. Although these agents are not first-line therapy for UC, multiple phase II and III trials have demonstrated checkpoint inhibitors’ significant roles in advanced/metastatic UC refractory to standard platinum-containing chemotherapy. Since 2016, the checkpoint inhibitors avelumab, atezolizumab, durvalumab, nivolumab, and pembrolizumab have received FDA (Food and Drug Administration) approval as treatment agents for bladder cancer (17). However, as of 2021, two of these agents, atezolizumab and durvalumab, have since been withdrawn (18, 19). PD-L1 expression is a potential predictive biomarker for PD-1/PD-L1 immunotherapy efficacy, but this connection is not yet clearly established. For example, some malignancies demonstrate higher responses to immunotherapy with greater PD-L1 expression, whereas other tumors’ responses to treatment have an inverse relationship with their PD-L1 expression. Additionally, the use of immunohistochemistry to quantify PD-L1 expression is limited by variation in tissue preparation and processing steps. There are also challenges in differentiating stained tumor cells from immune cells, primary tumor biopsies versus metastatic ones, and oncogenic PD-L1 expression versus induced expression (20). The timeline of FDA approvals for novel UC therapies from 2015 to 2021 is presented in **Table 1**.

**Table 1:** FDA approval timeline for novel UC therapies from 2015 to 2021.

Name of Drug	Class	FDA Approval Date	Studies Supporting Approval
Atezolizumab	PD-L1 inhibitor	5/2016 (for advanced or metastatic UC that worsened during/after platinum chemotherapy), 6/2018 (for cisplatin-ineligible advanced or metastatic UC with high PD-L1 expression, or for advanced/metastatic UC ineligible for any platinum therapy), withdrawn 3/2021	Rosenburg et al. (21), Necchi et al. (22), Galsky et al. (23)
Durvalumab	PD-L1 inhibitor	2/2017 (for advanced or metastatic UC that progressed during/after platinum), withdrawn 2/2021	Massard et al. (24), Powles et al. (25)
Nivolumab	PD-1 inhibitor	2/2017 (for advanced or metastatic UC that worsened during/after platinum)	CheckMate trials (26–28)
Avelumab	PD-L1 inhibitor	5/2017 (for advanced or metastatic UC that progressed during/after platinum or within 1 year of adjuvant/neoadjuvant platinum), 6/2020 (for maintenance treatment of advanced/metastatic that has not progressed after platinum)	Apolo et al. (29), Patel et al. (30), JAVELIN Bladder 100 (NCT02603432)
Pembrolizumab	PD-L1 inhibitor	5/2017 (for advanced or metastatic UC that progressed during/after platinum), 6/2018 (for cisplatin-ineligible advanced or metastatic UC with high PD-L1 expression, or for advanced/metastatic UC ineligible for any platinum therapy), 1/2020 (for high-risk BCG-unresponsive NMIBC)	KEYNOTE trials (31–35)
Erdafitinib	FGFR inhibitor	4/2019 (for advanced or metastatic UC with FGFR 2 or 3 that progressed after platinum)	Bahleda et al. (36), Loriot et al. (37)
Enfortumab vedotin	ADC targeting Nectin-4	12/2019 (for advanced or metastatic UC that progressed after two prior therapies)	Rosenburg et al. (38, 39)
Sacituzumab govitecan	ADC targeting Trop-2	4/2021 (for advanced or metastatic UC previously treated with platinum or PD-1/PD-L1)	Faltas et al. (40), TROPHY trial (NCT03547973)

The novel FDA-approved therapies for the treatment of UC since 2015 are listed here in chronological order of approval, with the supporting literature referenced for each. Atezolizumab and durvalumab were both withdrawn in early 2021.

Nivolumab targets the PD-1 receptor and was approved by the FDA in February 2017 (41). In the CheckMate 032 trial, a 2016 phase I/II study, nivolumab monotherapy in recurrent, metastatic UC produced an ORR of 24.4% (26). The next year, Sharma et al. published the CheckMate 275 trial, a phase II study involving nivolumab monotherapy in patients with metastatic or surgically unresectable UC that was refractory to platinum-based therapy. 265 patients were treated with nivolumab, and 52 patients (19.6%) achieved objective response. Although there was a higher ORR in the patient groups with a higher percentage of PD-L1 expression, significant clinical improvement was observed in all groups regardless of PD-L1 expression. 48(18%) experienced grade 3-4 adverse events (AE), the most common being grade 3 fatigue and diarrhea (27). A 2019 global analysis of the CheckMate 275 trial reached the same conclusions as those found in the original trial publication (42). In the adjuvant setting, the CheckMate274 phase 3, randomized, double-blind, multicenter trial compared 353 patients in nivolumab arm and 356 patients in the placebo arm among patients with high-risk muscle-invasive urothelial carcinoma (with primary tumor sites including bladder, ureter, or renal pelvis) after radical surgery. Patients were allowed but not required to have received neoadjuvant cisplatin. At 20 months median follow up, median disease-free survival was significantly longer for patients receiving nivolumab at 21 months compared to placebo at 11 months (HR: 0.70, 95% C.I. 0.54-0.89). A similar effect was observed in the PD-L1 ≥ 1% population where percentage of patients was 74.5% and 55.7%, respectively (HR 0.53, 95% C.I. 0.34-0.84). Grade 3 or 4 treatment-related adverse events occurred in 17.9% and 7.2% of patients in the nivolumab and placebo arms, respectively (43).

Avelumab is a monoclonal IgG antibody that targets PD-L1. It received accelerated approval by the FDA in May 2017 for treatment of locally advanced/metastatic UC that progressed during or after platinum-containing chemotherapy or within 1 year of receiving neoadjuvant or adjuvant platinum-containing chemotherapy (44). Avelumab then received approval in June 2020 for maintenance therapy in patients with locally advanced or metastatic UC that did not progress after first line platinum-containing chemotherapy (45). Phase I studies have demonstrated that avelumab is well-tolerated in patients with refractory, metastatic UC (29, 30). There is a randomized multicenter phase II study in the recruitment stage that compares the treatment outcomes of gemcitabine and cisplatin (GC) versus GC plus avelumab in patients with advanced UC. 90 patients are stratified by Karnofsky performance status and metastasis (visceral or non-visceral) (46). The recent JAVELIN Bladder 100 phase III trial compared the outcomes of avelumab with best supportive care (BSC) versus BSC alone for maintenance treatment of advanced/metastatic UC that did not worsen from first-line chemotherapy. The median OS in all patients, regardless of PD-L1 status in the tumors, was 21.4 months in the arm receiving avelumab, compared to an OS of only 14.3 months in the BSC arm (HR 0.69, CI 0.40 to 0.79, P < 0.001). In the overall population, the median PFS in the avelumab group was 3.7 months, compared to only 2.0 months in the control (HR 0.62, CI 0.52 to 0.75). In the PD-1 positive population, the median PFS was 5.7 months in the avelumab group and 2.1 months in the control (HR 0.56, CI 0.43 to 0.73). 11.9% of patients in the avelumab arm had treatment discontinued due to AEs, and the most common AEs grade 3 or greater in the avelumab group were UTI and anemia. These results demonstrated avelumab’s powerful role as first-line maintenance therapy for advanced UC, thus establishing a new standard of care for this type of UC (47). The stratified analysis found that there was a survival benefit with avelumab over the control group in all of the subgroups, regardless of whether the patients had first received cisplatin with gemcitabine or carboplatin with gemcitabine (48).

Pembrolizumab blocks PD-1, and in 2017 received accelerated FDA approval for patients with advanced/metastatic UC who are ineligible for cisplatin-containing chemotherapy. In 2018, the FDA modified this indication so that pembrolizumab could be used only in cisplatin-ineligible patients and high PDL1 expression with CPS (Combined Positive Score) >10, or in patients who are unfit for any platinum therapy, regardless of their PD-L1 expression as the KEYNOTE-361 trial showed decreased survival in patients with PD-L1-low status in the pembrolizumab monotherapy arm (49, 50). It also received regular FDA approval for advanced/metastatic UC progression during/after first-line platinum chemotherapy or within 12 months of receiving adjuvant/neoadjuvant platinum chemotherapy (51). Pembrolizumab’s accelerated approval was based upon the phase II KEYNOTE-052 trial’s data, which treated 370 advanced/metastatic UC patients who were not eligible for cisplatin-based chemotherapy; median follow-up was 5 months, and the ORR was 28.6% (31, 51). Vuky et al.’s 2020 paper examined the long-term outcomes of the KEYNOTE-052 patients, with a minimum follow-up time of 2 years since the last patient was enrolled. The ORR was 28.6%, and the median response duration was 30.1 months. 33 (8.9%) of patients had complete response, and 73 (19.7%) achieved partial response (52). Regular approval was given based upon the results of the phase III KEYNOTE-045 trial, where 542 patients with advanced UC that progressed or recurred following platinum-based chemotherapy were assigned to receive either pembrolizumab or the investigator’s choice of paclitaxel, docetaxel, or vinflunine. The median OS was 10.3 months in the group receiving pembrolizumab, compared to 7.4 months in the chemotherapy group (HR 0.73, CI 0.59 to 0.91, P = 0.002). There were no significant differences in the duration of PFS between the two treatment arms (HR 0.98, CI 0.81 to 1.19, P = 0.42) (51, 53). However, the phase III KEYNOTE-361 trial involved 1010 patients with untreated advanced/metastatic or unresectable UC, with an Eastern Cooperative Oncology Group (ECOG) group score of 2 or lower and did not find a significant survival benefit with pembrolizumab regimens. Patients were randomly assigned to receive either pembrolizumab with gemcitabine, pembrolizumab alone, or chemotherapy alone. The median PFS in the pembrolizumab with chemotherapy group was 8.3 months versus 7.1 months in the chemotherapy only group (HR 0.78, CI 0.65 to 0.93, P = 0.0033). The median OS was 17.0 months in the pembrolizumab with chemotherapy arm, versus 14.3 months in the chemotherapy arm (HR 0.86, CI 0.72 to 1.02, P = 0.0407). The most common grade 3-4 AE was anemia (104 [30%] of 349) in the pembrolizumab and chemotherapy group. In the pembrolizumab only arm, diarrhea, fatigue and hyponatremia were the most frequent grade 3-4 AEs, each occurring in 4 (1%) of the 302 patients (50).

Atezolizumab targets PD-L1 and was initially FDA-approved in May 2016 for locally advanced/metastatic UC that worsened during or after platinum-based chemotherapy, or within 1 year of platinum-based chemotherapy, before or after surgical intervention (54). In 2018, the FDA modified this indication to be used either for patients ineligible for cisplatin with PDL-1 stained tumor infiltrating cells >5%, or for patients ineligible for any platinum-based therapy, regardless of PD-L1 expression (49). However, in March 2021, Roche voluntarily withdrew the FDA indication for atezolizumab for advanced/metastatic UC previously treated with platinum-based therapy (18). Rosenburg et al.’s 2016 phase II trial administered atezolizumab to 310 patients who had locally advanced or metastatic UC refractory to platinum-based chemotherapy. The treatment was appropriately tolerated, with an overall response rate of 15%, regardless of PD-L1 expression status of the infiltrating immune cells (21). Additionally, the IMvigor 210 study published in 2017 found that UC patients who continued atezolizumab therapy after RECIST v.1.1 progression occurred had a median overall survival (OS) of 8.6 months, compared to a median post-progression OS of 6.8 months in those receiving an alternative treatment (22). The phase III IMvigor 211 trial involved patients with metastatic UC that progressed after platinum-based therapy, who were randomly assigned to receive either atezolizumab or the investigator’s choice of chemotherapy (vinflunine, paclitaxel, or docetaxel). The study’s primary endpoint of improved OS in patients with PD-L1 positive tumors was not achieved (55). The IMvigor130 trial, a multicenter phase III study published in 2020, involved 1213 patients with advanced/metastatic UC. Patients were randomly placed into one of three treatment groups: atezolizumab with platinum-based chemotherapy, atezolizumab alone, or platinum-based chemotherapy with a placebo. In the combination therapy group, median PFS was 8.2 months (CI 6.5 to 8.3), compared to 6.3 months (CI 6.2 to 7.0) in the chemotherapy only group (HR 0.82, CI 0.70 to 0.96, P = 0.007). The median OS in the combination therapy arm was 16 months (CI 13.9 to 18.9), versus 13.4 months (CI 12.0 to 15.2) in the chemotherapy only arm (HR 0.83, CI 0.69 to 1.00, P = 0.027). The combination therapy arm had a safety profile similar to atezolizumab or platinum-based chemotherapy used alone (23). Additionally, in the spring of 2021 the FDA panel has voted for continued support of the accelerated approval of atezolizumab as frontline treatment in cisplatin-ineligible advanced/metastatic UC until the final analysis of the IMvigor 130 trial is released in late 2022 (56). Additionally, the phase III IMvigor 010 trial studied adjuvant atezolizumab therapy in MIBC patients with ECOG score 0-2 and high-risk of recurrence after primary resection. However, the endpoint of disease-free survival was not met. Of the 390 patients in the atezolizumab arm, the most common grade 3-4 AEs were urinary tract infection [31(8%)], pyelonephritis [12(3%)], and anemia [8(2%)] (57).

Durvalumab is another human monoclonal antibody that binds to PD-L1, initially approved by the FDA for advanced UC in February 2017 (58). In a 2017 phase I/II open-label study, Powles et al. assessed the safety profile and efficacy of durvalumab in patients with either locally advanced or metastatic UC. Among 191 patients, the ORR was 17.8%, 3.7% of patients achieved CR, overall survival was 18.2 months, and the median PFS was 1.5 months. 6.8% of patients experienced grade 3-4 treatment-related adverse effects (25). The most common grade 3-4 AEs in the overall population receiving durvalumab were fatigue [16(1.6%) out of 970 patients], elevated AST [11(1.1%)], and elevated GGT [8(0.08%)]. There is also a phase II trial in the recruitment phase that will examine safety and efficacy of dose dense methotrexate, vinblastine, doxorubicin, and cisplatin (ddMVAC) in combination with the checkpoint inhibitors durvalumab and tremelimumab, which targets CTLA-4 rather than PD-L1, for patients with muscle-invasive UC (59). However, like atezolizumab, durvalumab was withdrawn by its developer AstraZeneca as therapy for locally advanced or metastatic UC refractory to chemotherapy in February 2021 (60). The phase III trial DANUBE compared durvalumab with or without tremelimumab versus first-line chemotherapy for stage IV UC but failed to reach its primary end point of OS. The most common grade 3-4 AE was elevated lipase in the durvalumab-only group [7(2%) out of 345 patients] and in the durvalumab with tremelimumab group [16(5%) of 313] (61).

Ipilimumab is a CTLA-4 inhibitor that has not yet been FDA-approved for UC therapy; however, it is a promising therapy when combined with another checkpoint inhibitor such as nivolumab. Sharma et al.’s 2019 multicohort study CheckMate 032 suggested that combination treatment with ipilimumab and nivolumab is a safe regimen that produces greater antitumor outcomes than if either of these therapies were given alone. Patients with metastatic or locally advanced UC were randomized to either a nivolumab 3 mg/kg per 2 weeks monotherapy group (N3), nivolumab 3 mg/kg with ipilimumab 1 mg/kg every 3 weeks for 4 doses followed by nivolumab monotherapy 3 mg/kg every 2 weeks (N3I1), or nivolumab 1 mg/kg with ipilimumab 3 mg/kg every 3 weeks for four doses followed by nivolumab monotherapy 3 mg/kg every 2 weeks (N1I3). The ORRs in the N3, N3I1, and N1I3 arms were 25.6%, 26.9%, and 38.0% respectively. The median response duration was greater than 22 months in all arms. Across all of the treatment arms, the most common therapy-related AEs were fatigue, elevated AST and ALT, pruritis, rash, diarrhea, and reduced appetite. Grade 5 immune-related AE occurred only in the N3 and N3I1 arms (28). In the neo-adjuvant setting, the NABUCCO trial published in 2020 treated 24 stage III UC patients with two doses of ipilimumab then two doses of nivolumab before resection of the malignancy. 23 of the 24 patients achieved the primary endpoint of feasibility to resect within 12 weeks of initiating treatment, and 11 (46%) patients showed a complete pathological response, the secondary endpoint (62). The long-term follow-up data also demonstrate the N1I3 arm’s therapeutic promise. The median OS in the N1I3 group was the longest at 15.3 months, compared to 9.9 months in the N3 group and 7.4 months in the N3I1 group. The N3I1 also produced the greatest ORRs per both investigator review and blinded independent review (42% and 38%, respectively) (63). The ongoing phase III CheckMate 901 trial is studying the outcomes in previously untreated UC patients who are given the N1I3 regimen or standard of care chemotherapy (NCT03036098) (64).

### Targeting FGFR

The FGF/FGFR pathway is involved in the development of various malignancies, affecting the major signaling pathways for tumor proliferation (**Figure 1**). Some therapeutic agents are nonselective, targeting all FGF receptors, whereas others are more selective. Erdafitinib gained FDA approval as therapy for locally advanced or metastatic UC with an FGFR2 or FGFR3 mutation in April 2019 (65). Erdafitinib is a FGFR1-4 inhibitor that has a manageable safety profile in patients with advanced tumors such as UC (36). A phase II trial published in 2019 found that erdafitinib produced a 40% ORR in patients with metastatic UC who progressed after least one course of chemotherapy or 12 months of adjuvant or neoadjuvant chemotherapy and possibly immunotherapy, with a median PFS of 5.5 months and median OS of 13.8 months. Out of the 99 patients, the most common grade ≥3 AEs included hyponatremia (11%), stomatitis (10%), asthenia (7%), nail dystrophy (6%), and urinary tract infection (5%) (37).

### Enfortumab Vedotin

Enfortumab vedotin (EV) gained FDA approval in December 2019 for the treatment of locally advanced or metastatic UC in patients who had already received a PD-1 or PD-L1 inhibitor and adjuvant or neoadjuvant platinum-containing chemotherapy (66). It is an antibody drug conjugate (ADC) that targets the adhesion molecule Nectin-4, which is expressed on many UC cells (**Figure 1**). After binding to Nectin-4, the ADC is internalized by the tumor cell and induces cytotoxic effects *via* disruption of microtubule function, which is accomplished by monomethyl auristatin E, the drug that is conjugated to the antibody (67). The phase I trial EV-101 administered single-agent EV to patients with solid malignancies expressing Nectin-4, including metastatic UC. The therapy was well tolerated, the ORR was 43% with a median OS of 12.3 months, and OS was 51.8% at 1 year (38). In a phase II single-arm EV-201 trial involving patients with advanced/metastatic UC previously treated with platinum chemotherapy and PD-1 or PD-L1 inhibitors, EV therapy produced an ORR of 44% with 12% having CR, median response duration of 7.6 months, and minimal adverse treatment related events (39). The phase III EV-301 trial compared the outcomes of patients treated with EV versus chemotherapy (investigator’s choice of docetaxel, paclitaxel, or vinflunine) following platinum and checkpoint inhibitor therapy. The OS in the EV group was 12.88 months, compared to only 8.97 months in the chemotherapy group (HR 0.7, CI 0.56 to 0.89, P = 0.001). The EV group also had a greater PFS than the chemotherapy group (5.55 months versus 3.71 months, HR 0.62, CI 0.51 to 0.75, P < 0.001). Among 296 patients in the EV arm, the most common grade ≥3 AEs were maculopapular rash [22(7.4%)], fatigue [19(6.4%)], and reduced neutrophil count [18(6.1%)] (68). The ongoing phase III EV-302 trial will compare the outcomes of previously untreated UC patients who are given either EV with pembrolizumab or gemcitabine with platinum-based chemotherapy (NCT04223856).

### Antibody-Drug Conjugates

Sacituzumab govitecan (IMMU-132) is an antibody-drug conjugate containing SN-38, a metabolite in irinotecan which inhibits the activity of topoisomerase (**Figure 1**). In a phase I trial, 6 patients with metastatic UC refractory to platinum therapy were treated with IMMU-132. This agent was well-tolerated overall, and three patients had significant response, with a PFS 6.7-8.2 months, and OS 7.5-11.4 months (40). Phase II of this trial was completed in April 2021 (NCT01631552). Additionally, the phase II TROPHY trial administered IMMU-132 to locally advanced or metastatic UC who previously received platinum-based chemotherapy and either a PD-1 or PD-L1 inhibitor. The ORR was 27.7%, and median duration of response of 7.2 months (NCT03547973). In April 2021, sacituzumab govitecan received accelerated FDA approval for treatment of advanced UC (69).

### Targeting Angiogenesis

VEGF receptors play a prominent role in tumor growth and have been studied as combination therapy with platinum-based chemotherapy for advanced UC. Although ramucirumab is not FDA-approved for the management of UC, it may provide a promising alternative to standard chemotherapy regimens in the future. A phase III randomized, double-blind trial administered ramucirumab in combination with placebo or IV docetaxel therapy for patients with advanced/metastatic UC who progressed during or after platinum-based therapy. The PFS in the ramucirumab plus docetaxel group was 4.07 months (HR 0.696, CI 0.573 to 0.845, P = 0.0002), which was significantly longer than the PFS of 2.76 months in the docetaxel only group. Median OS was 9.4 months in the combination therapy group compared to only 7.9 months in the docetaxel only group (HR 0.887, CI 0.724 to 1.086, P = 0.25). The most common grade 3 or greater AEs in the ramucirumab with docetaxel group were febrile neutropenia [24(9%) out of 258 patients], reduced neutrophil count [23(9%)], fatigue [17(7%)], hypertension [11(4%)], and diarrhea [8(3%)]. Although these results show promise for ramucirumab in the future for advanced UC management, the generalizability of this study is limited by its particular inclusion and exclusion criteria. For example, eligible patients must have had disease progression according to the Response Evaluation Criteria in Solid Tumors (RESIST) within 14 months or less after receiving platinum-based chemotherapy. The investigators allowed patients who had previously been treated with one checkpoint inhibitor to be included in the study, so long as they had relapsed within 24 months or receiving a platinum-containing treatment plan. Patients who had received more than one treatment of systemic chemotherapy for relapsed or metastatic UC were not eligible (70).

## Novel Molecular Targets for Management for Non-Muscle Invasive Bladder Cancer

### Immunotherapy

In January 2020, pembrolizumab also gained FDA approval as treatment for NMIBC with carcinoma *in situ* that is unresponsive to Bacillus Calmette-Guerin (BCG) (71). The phase II KEYNOTE-057 trial (NCT02625961) administered pembrolizumab monotherapy in high-risk NMIBC (T1, high grade Ta and/or carcinoma *in situ* [CIS] only) patients, involving one cohort of CIS patients with or without papillary tumors that failed BCG therapy. The published results of the cohort indicate that pembrolizumab has significant activity, with an appropriate safety profile. The CRR at 3 months was 38.8%. In 80.2% of the patients, CR was durable at 6 months, and grade 3 or 4 treatment related AEs only occurred in 12.6% of the cohort. The CRRs were 44.6%, 41.7%, and 28.0% for patients with CIS alone, CIS with T1 tumors, and CIS with high-grade Ta tumors, respectively. 75% of patients demonstrated complete response for ≥ 6 months, and 53% for ≥ 9 months. Out of 101 patients, the most common grade 3-4 treatment related AEs were arthralgia (2%) and hyponatremia (3%) (32). The KEYNOTE-676 trial, which will study the efficacy of pembrolizumab with BCG treatment in high-risk NMIBC patients, is currently in the enrollment phase (NCT03711032).

### Immunomodulators

Keyhole limpet hemocyanin (KLH) is a large carrier protein that can carry multiple tumor antigens to stimulate significant antibody production. Lammers et al. conducted a phase III trial comparing the outcomes of immunotherapy with adjuvant KLH versus immunotherapy with mitomycin C. Among the NMIBC patients who received KLH, 61% developed recurrence, whereas only 34% of those who received MM had recurrence. Median RFS in the KLH group compared to the MM group was 106 weeks versus 297 weeks (approaching significance P = 0.05, log-rank test). The majority of the AEs in both arms were mild, with fever, fatigue, and flu-like symptoms occurring more frequently in the KLH group (72). However, a 2017 retrospective analysis of this trial argues that outcomes from each drug regimen may have been skewed by operator dependence of the surgeons performing the patient population’s TURBT; thus, further trials must be conducted to elucidate the efficacy of KLH (73).

### Gene Therapy

Nadofaragene firadenovec is an intravesical therapy comprised of a recombinant adenovirus that delivers the human interferon alfa-2b gene to the bladder epithelial cells. A phase III involved 151 BCG-nonresponsive NMIBC patients, who each received at least one dose of intravesical nadofaragene firadenoec. Additional doses were given at month 3, 6 and 9 if there was no high-grade recurrence. Out of 103 patients with carcinoma in situ, 55 (53.4%) had complete response within 3 months of receiving the first dose, and 25 out of the 55 patients (45.5%) showed maintained response at 12 months. Only 6 (4%) patients experienced grade 3 adverse events, the most common being was micturition urgency, occurring in 2 patients. No grade 4-5 events occurred. Although these results demonstrated the treatment efficacy of nadofaragene firadenovec and the need for further research on this agent, this study was limited because there was no central pathology review and initial responses were gauged by cystoscopy and cytology reports, which are contingent upon the experience and criteria utlized by the individual pathologist (74).

## Future Perspectives for Bladder Cancer Treatment

There are multiple phase I and phase II trials (ongoing or recently completed) and ongoing phase III trials studying diverse therapies for both advanced or metastatic bladder cancer and NMIBC. The trials and the studied agents are listed in **Table 2**.

**Table 2:** Emerging therapies for advanced/metastatic UC or NMIBC.

Novel Agent	Primary Mechanism	UC Population Treated	Trial Phase	Study/Trial #
Toripalimab	PD-1 antibody	Advanced UC	I	Tang et al. (75)
Nivolumab with concomitant radiation	PD-1 antibody	Advanced UC that will undergo cystectomy	II	Schmid et al. (76)
Tremelimumab + Durvalumab	CTLA-4 antibody PD-1 antibody	Advanced UC	II	NCT02527434
Enfortumab vedotin + Atezolizumab	Antibody-drug conjugate PD-L1 ligand	Advanced/metastatic UC	Ib/II	NCT03869190
Enoblituzumab	B7-H3 antibody	Refractory UC	I	NCT01391143
Tislelizumab	PD-1 antibody	Advanced/metastatic UC	II	NCT04004221
Rogaratinib	Pan-FGFR inhibitor	Advanced UC	I I	Schuler et al. (77) NCT01976741
Derazantanib	Pan-FGFR inhibitor	Advanced UC	I	Papadopoulos (78)
Infigratinib	Pan-FGFR inhibitor	Advanced UC	I	NCT01004224
Carotuximab	Endoglin antibody	Advanced/metastatic UC	II	Apolo et al. (79)
Axitinib + Avelumab	VEGFR inhibitor PD-L1 antibody	Advanced/metastatic UC (cisplatin ineligible)	II	NCT03472560
Ramucirumab	VEGFR inhibitor	Advanced UC refractory to prior therapy	I	Herbst et al. (80)
Tisotumab vedotin	Antibody-drug conjugate	Advanced/metastatic UC	I/II	De Bono et al. (81)
Ado-trastuzumab	Antibody-drug conjugate	Advanced UC	I	NCT02675829
Afatanib	EGFR/HER2 inhibitor	Metastatic UC after platinum failure	II	Choudhury et al. (82)
			II	NCT02122172
			II	NCT02780687
Genestein	EGFR inhibitor	Advanced UC that will undergo cystectomy or TURBT	II	NCT00118040
Everolimus	mTOR inhibitor	Metastatic UC progressing after 1-4 cytotoxic agents	II	Seront et al. (83)
		Metastatic UC after platinum failure	II	Milowsky et al. (84)
Temsirolimus	mTOR inhibitor	Recurrent/metastatic UC after platinum	II	Pulido et al. (85)
Cabozantanib	Multikinase inhibitor	Metastatic UC refractory to platinum	II	Apolo et al. (86)
Sorafenib	Multikinase inhibitor	Metastatic UC that progressed after platinum	I	Shah et al. (87)
Pazopanib + Everolimus	Multikinase inhibitor mTOR inhibitor	Metastatic UC ineligible for platinum or progressed after platinum	I	Bellmunt et al. (88)
Regorafenib	Multikinase inhibitor	Metastatic UC that progressed after platinum	II	NCT02459119
Merestinib	Multikinase inhibitor	Advanced/metastatic UC	I	NCT03027284
Selumetinib	Multikinase inhibitor	Advanced/metastatic UC that progressed with prior therapy	Ib	NCT02546661
Vistusertib	MTOR inhibitor			
AZD4547	FGFR 1/2/3 inhibitor			
Adavosertib	WEE1 inhibitor			
Olaparib	PARP inhibitor			
Durvalumab	PD-L1 inhibitor			
SGT-94	RB94 plasmid	Metastatic UC with no available standard therapy	I	Siefker-Radke et al. (89)
Docetaxel + Apatorsen	Microtubule inhibitor Hsp27 inhibitor	Metastatic UC that relapsed after platinum	II	Rosenberg et al. (90)
Paclitaxel	Microtubule inhibitor	Advanced UC that progressed after cisplatin	II	Ogawa et al. (91)
		Advanced UC after platinum and pembrolizumab failed		Furubayashi et al. (92)
		Metastatic UC after cisplatin failed		Kobayashi et al. (93)
Cabazitaxel	Microtubule inhibitor	MIBC eligible for cystectomy	II	Challapalli et al. (94)
Pemetrexed	Folate antimetabolite	Advanced UC resistant to platinum	N/A	Bambury et al. (95)
		Advanced UC	II	Choi et al. (96)
		Advanced/metastatic UC	I	Cao et al. (97)
		Metastatic UC refractory to platinum	I	Pappot et al. (98)
Apatorsen	Hsp27 inhibitor	Advanced, untreated UC	II	Bellmunt et al. (99)
Pralatrexate + Palbociclib	DHFR inhibitor CDK 4/6 inhibitor	Advanced UC	N/A	Wang et al. (100)
Flouorocyclo- -pentenyl cytosine	Cytidine analog and methyltransferase inhibitor	Advanced UC	IIa	Adashek et al. (101), NCT02030067
Olaparib + Durvalumab	ADP ribose polymerase inhibitor PD-L1 inhibitor	Unresectable metastatic UC ineligible for platinum	II	NCT03459846
		Resectable metastatic UC to undergo surgery	II	Rodriguez-Moreno et al. (102), NCT03534492
FyCyd + THU	Methytransferase inhibitor Cytidine deaminase inhibitor	Unresectable or metastatic UC that progressed after platinum	II	NCT00978250
CAR-T cells	Target specific tumor antigens	Relapsed and refractory UC	I	NCT03960060
PF-04518600 + Utomilumab	OX40 agonist 4-1BB agonist	Advanced/metastatic UC	I	NCT02315066
TAEST16001	Target specific tumor antigens	Metastatic UC that failed multi-line treatment	I	NCT03159585
Cytokine induced killer cells	Immune system stimulation	Advanced UC	II	NCT02489890
Personalized peptide vaccination	Induce tumor immunity	Progressive advanced UC	I/II	Obara et al. (103)
		Advanced/metastatic upper tract UC after platinum failure	II	Suekane et al. (104)
		Metastatic UC refractory to platinum	II	Noguchi et al. (105)
		Advanced/metastatic UC	I	NCT02897765
Modified vaccinia Ankara virus + Pembrolizumab	Augment host immune response PD-1 inhibitor	Unresectable metastatic UC that failed prior therapy	I	NCT02432963
CAVATAK	Oncolytic virus	Advanced/metastatic UC	I	NCT02043665
Navoximod + Atezolizumab	IDO1 inhibitor PD-L1 inhibitor	Advanced UC	Ib	Jung et al. (106)
Epacadostat	IDO1 inhibitor	Advanced/metastatic UC	I/II	NCT02178722
INCB001158 + Pembrolizumab	Arginase inhibitor	Advanced/metastatic UC	I/II	NCT02903914
Pirarubicin	DNA intercalator and topoisomerase inhibitor	NMIBC after TURBT	N/A	Huang et al. (107)
Cabazitaxel	Microtubule inhibitor	NMIBC that failed BCG	I	DeCastro et al. (108)
Apaziquone	DNA crosslinker	NMIBC after TURBT	III	NCT03224182
			III	NCT02563561
Vicinium	Protein synthesis inhibitor	NMIBC in situ, or high grade papillary bladder cancer that failed BCG	III	NCT02449239
Dendritic cells	Induce immune response	3 NMIBC + 2 metastatic UC patients	I/II	Ogasawara et al. (109)
CAVATAK	Oncolytic virus	NMIBC scheduled for TURBT	I	Annels et al. (110)
Enadenotucirev	Oncolytic virus	NMIBC scheduled for TUBRT	I	Garcia-Carbonero et al. (111)
CG0070	Oncolytic virus	High-grade NMIBC after BCG failure	II	NCT02365818
PANVAC	Induce tumor immunity	High-grade NMIBC after BCG failure	I	NCT02015104
Personalized peptide vaccination	Induce tumor immunity	NMIBC after TURBT	I	Obara et al. (112)
MAGE-A3	Induce tumor immunity	NMIBC after TURBT	I	Derre et al. (113)
Infigratinib	FGFR-3 inhibitor	NMIBC after prior BCG therapy	N/A	NCT02657486
Rapamycin (sirolimus)	mTOR inhibitor	NMIBC after BCG failure	I/II	NCT02009332
		NMIBC	II	NCT04375813
APL-1202	Methionine aminopeptidase II inhibitor	NMIBC resistant to one induction BCG course	Ib	NCT03672240
Nadofaragene firadenovec	Interferon-alpha transmitted to tumor environment	High-grade NMIBC refractory to BCG or relapsed	II	Shore et al. (114)
			III	NCT02773849
Imiquimod	Toll-like receptor 7 agonist	NMIBC *in situ*	I	Arends et al. (115)
			II	Donin et al. (116)

The emerging therapeutic agents for UC with completed or ongoing phase I or II or incomplete III trials with novel molecular targets are listed below. To highlight the novel agents for UC management, standard chemotherapy agents involved in some studies were not included in this table.

### Phase I and II Trials for Advanced/Metastatic UC Treatment

#### Immunotherapy

PD-1/PD-L1 and CTLA-4 inhibitors continue to be investigated for advanced/metastatic UC therapy. Toripalimab is a humanized antibody targeting PD-1, was studied in a phase I trial published in 2019 and showed promising results. 36 patients with either advanced UC, renal cell carcinoma (RCC), or melanoma resistant to first-line therapy were given toripalimab, with an average of 12.4 doses. The therapy was well-tolerated, with most of the treatment related adverse events being grade 1 or 2. The most common grade 3-4 AEs were elevated lipase and anemia (75).

In an ongoing phase II trial, nivolumab is being studied as preoperative therapy concomitantly with radiation, in patients with locally advanced bladder cancer who then undergo radical cystectomy with lymphadenectomy (NCT03529890). Nivolumab 240 mg will be administered for a total of 4 cycles, every 2 weeks, with concomitant radiation therapy. The primary endpoint will be the rate of patients who completed the regimen (radiation with nivolumab, followed by radical cystectomy) after week 15 (76).

Tremelimumab is an CTLA-4 inhibitor that can potentially contribute to UC management. There is an ongoing phase II trial comparing tremelimumab monotherapy, durvalumab monotherapy, and durvalumab and tremelimumab combination therapy for advanced solid tumors, including UC. The most recent results update show that there was a 6.2% complete response rate in the advanced UC arm. Out of 32 UC patients, 18 had severe adverse events, with diarrhea and colitis being the most common (NCT02527434).

Another phase Ib/II study in the recruitment phase will compare the outcomes of various immunotherapy-based treatment combinations in patients with locally advanced or metastatic UC, involving drugs such as EV and atezolizumab (NCT03869190).

Enoblituzumab is a monoclonal antibody that targets B7-H3, a checkpoint protein that promotes tumor proliferation and regulates the metabolic activity of cancer cells. It is being studied in a phase I trial for patients with refractory cancer, including malignancies such as bladder cancer (NCT01391143).

Tislelizumab is a PD-1 monoclonal antibody that is currently being studied in a phase II trial, involving patients with advanced or metastatic UC who had progressed after platinum-based chemotherapy (NCT04004221).

#### Targeting FGFR

Rogaratinib is another oral pan FGFR inhibitor that was studied in a phase I trial involving patients with various advanced malignancies, including UC. Out of the 126 patients treated, 52 of them were UC patients whose malignancy overexpressed the mRNA for FGFR. The agent was well-tolerated, and 15% of the patients in the expansion cohorts achieved an objective response. The most common treatment-related AEs were hyperphosphatemia [77(61%) out of 126 patients] and diarrhea [65(52%)] (77). A recently completed phase I study involving 168 patients with metastatic or advanced malignancies such as UC also found that rogaritinib has a manageable safety profile in this population (NCT01976741).

The pan FGFR inhibitor derazantinib (ARQ 087) may also have a promising role in advanced UC treatment. The first human study involving derazantinib was published in 2017, and this phase I trial had a manageable safety profile in patients with advanced solid tumors. The most common treatment-related AEs were fatigue, nausea, and increased AST. 14 out of 80 patients (18%) experienced AEs grade ≥3 (78).

Infigratinib (BGJ398) is a pan-FGFR inhibitor that is being studied in bladder cancer patients. There an ongoing phase I dose escalation study examining the safety and effects of infigratinib in patients with advanced solid malignancies including UC (NCT01004224).

#### Targeting Angiogenesis

Multiple trials are investigating agents targeting the angiogenesis pathway. Carotuximab (TRC105) is a monoclonal antibody that binds to endoglin (CD105), a membrane glycoprotein that is part of the TGF beta receptor, which helps regulate angiogenesis. A phase II trial administered TRC105 to patients with previously treated advanced UC, and found that, although well-tolerated, TRC105 did not improve 6-month survival in these patients. The group’s 3-month PFS probability was 18.2%, with a median OS of 8.3 months. Among the 13 patients enrolled, the most common AEs were headache (9[69%]) and infusion-related reactions (9[69%]). Only 2 patients experienced grade ≥3 AEs (79).

Axitinib inhibits VEGF receptors 1-3, and is being studied as combination therapy with avelumab in a phase 2 trial for patients with NSCLC who have received at least one platinum-containing therapy previously and patients with treatment naïve or metastatic/advanced UC who are ineligible for cisplatin. The primary outcome will be objective response based on RECIST version 1.1 criteria, and the predicted completion date is June 2022 (NCT03472560).

Ramucirumab is a VEGFR-2 antagonist, disrupting the angiogenesis pathway regulated by VEGF in tumor cells. A phase 1a/b trial studied the safety of ramucirumab with pembrolizumab therapy for patients with advanced gastroesophageal adenocarcinoma, NSCLC, or UC refractory to prior treatment. The study concluded that this combination therapy had a manageable safety profile; out of 92 patients, 22 patients had grade ≥3 treatment-related adverse events, with hypertension occurring in 6 patients and colitis occurring in 5 (80).

#### Antibody-Drug Conjugates

Tisotumab vedotin is an antibody-drug conjugate that targets tissue factor, which is expressed on most solid malignancies. A phase I/II study involving 147 patients with either relapsed or advanced/metastatic malignancies, including UC, found that tisotumab vedotin treatment produced an ORR of 15.6%, and 27% experienced rate of severe AEs, the most common grade ≥3 AEs being fatigue (14[10%]), anemia (8[5%]), abdominal pain (6[4%]), and hypokalemia (6[4%]) (81).

Ado-trastuzumab emtansine is an antibody-drug conjugate that allows trastuzumab, which binds to HER2 receptors, to be internalized by tumor cells. An ongoing phase II trial will assess the best overall response over a period of 2 years in patients with HER-2 mutant or amplified malignancies, including UC (NCT02675829).

#### Targeting EGFR, HER Pathways

Afatinib inhibits the EGFR, HER-2 and HER-4 tyrosine kinases. A phase II trial administered afatinib to 23 patients with metastatic UC refractory to platinum therapy. 5 (21.7%) patients achieved partial response or stable disease, and the median time before progression was 6.6 months in patients with HER2 amplifications or EGFR mutations. Out of the 23 patients, the most common grade 3 AEs were nausea and vomiting, diarrhea, acneiform rash, and fatigue (82). There is a phase II trial currently in the recruitment phase that will measure the 3-month PFS rate in patients with metastatic UC refractory to platinum therapy after receiving afatinib (NCT02122172). Another phase II trial will measure the 6-month PFS in advanced or metastatic UC patients who have failed platinum therapy and have HER-2, HER-3, or EGFR genetic alterations (NCT02780687).

Another phase II randomized, double arm trial is studying the phosphorylation activity of Genestein (G-2535) on EGF receptors in stage I to stage III non-invasive and invasive bladder cancer patients. Multiple tissue biomarkers will be compared between the placebo, high dose G-2535, and low dose G-2535 groups (NCT00118040).

#### Targeting the mTOR Pathway

There is also growing research supporting the use of agents targeting PI3K/AKT/mTOR, a signaling pathway which regulates tumor proliferation, for localized UC therapy. Everolimus is an oral mTOR inhibitor that has demonstrated clinical benefit in advanced/metastatic UC. Seront et al.’s 2012 phase II trial enrolled 37 patients with advanced UC that had failed platinum chemotherapy. 2 patients had partial response, and 8 achieved stable disease, with a disease control rate of 27% after 8 weeks. The most common grade 3-4 AEs were fatigue [10(27%)] and thrombopenia [5(13%)] (83). Additionally, Milowsky et al.’s 2013 phase II trial administered everolimus to 45 patients with metastatic UC that progressed after receiving cytotoxic therapy. After 2 months, 23 (51%) patients were progression free, the median PFS was 2.6 months and median OS was 8.3 months (95% CI). The most common grade 3-4 AEs included anemia [9(20%)], abnormal INR [7(16%)], and infection [6(13%)] (84).

Temsirolimus is an mTOR inhibitor that is being considered as potential second-line therapy for UC. A phase II trial evaluated patients with recurrent or metastatic bladder cancer treated with temsirolimus. The median PFS was 2.8 months and OS was 7.2 months. However, 50 of 53 patients experienced grade 1 or 2 treatment related toxicity, and 28 patients had a grade 3 or 4 adverse event, so this high risk of adverse events must be weighed carefully against its therapeutic impact. The most common grade 3-4 AEs were hematologic toxicity [10(18.9%)], asthenia/fatigue [10(18.9%)], and gastrointestinal toxicity [6(11.3%)] (85).

#### Kinase Inhibitors

Kinase inhibitors can bind to multiple tumor cell receptors and thereby inhibit major proliferation pathways. Cabozantinib inhibits multiple receptor tyrosine kinases such as VEGFR, RET, AXL, and MET, which regulate signaling cascades involved in tumor proliferation. In a phase II trial involving patients with metastatic UC refractory to platinum-based therapy, cabozantinib was well-tolerated and produced an ORR of 19%. Among 68 patients, the most common grade 3-4 AEs were fatigue [6(9%)], hypertension [5(7%)], proteinuria [4(6%)], and hypophosphatemia [4(6%)] (86).

Sorafenib is another multi-kinase inhibitor, targeting VEGFR, PDGFR, and many RAF kinases, whose safety and efficacy have been assessed in UC patients. A phase I trial administered sorafenib with vinflunine therapy to patients with metastatic UC that progressed following platinum-based chemotherapy. The median OS was 7.0 months, and the ORR was 41%. The most frequent grade 3-4 AEs were neutropenia (6 of 22 patients), febrile neutropenia (5), and hyponatremia (5) (87).

Additionally, a 2018 phase I trial studied the effects of everolimus with the tyrosine kinase inhibitor pazopanib in 19 metastatic UC patients, 3 NSCLC patients, and 1 adrenocortical carcinoma patient (all of whom had previously received their respective standard therapies, followed by disease progression) and produced promising results. The ORR was 21%, and 73.7% of patients experienced a grade 3 or higher treatment related toxicity. The most frequent grade 3-4 treatment-related AEs were fatigue, elevated AST, thrombocytopenia and hypophosphatemia (88).

The multi-kinase inhibitor regorafenib, which especially targets kinases involved in angiogenesis, was studied in a phase II trial for 17 patients with progressive, metastatic UC. The primary outcome, 6-month PFS, was 17.6%. Grade 3 AEs included diarrhea, fatigue, low phosphorous levels, anemia and thrombocytopenia (NCT02459119).

Merestinib inhibits multiple tyrosine kinases, including c-MET, which has high activity in multiple malignancies. A phase I trial is comparing the safety of merestinib monotherapy to merestinib combined with cisplatin and gemcitabine for advanced or metastatic malignancies such as UC (NCT03027284).

There is an ongoing phase Ib trial examining the safety of multiple unique agents for the treatment of MIBC that progressed following standard treatment. These agents include the multikinase inhibitor selumetinib, the mTOR inhibitor vistusertib, the PARP inhibitor Olaparib, the FGFR 1/2/3 inhibitor AZD4547, and the tyrosine kinase inhibitor adavosersitib, which targets Wee1, a cell cycle regulator that phosphorylates Cdk1 (NCT02546661).

#### Gene Therapy

Gene therapy can safely produce toxic effects on bladder cancer cells. SGT-94 carries a plasmid that encodes the truncated version of the retinoblastoma gene, which promotes tumor suppression. A phase I trial showed that SGT-94 was safe in patients with metastatic genitourinary cancers that had no available standard therapy, and did not affect normal urothelial cells. Only 2 patients experienced grade 3-4 adverse events (lymphopenia and neutropenia) (89).

#### DNA Replication/Repair/Gene Expression/Protein Synthesis Inhibition and Taxanes

A handful of therapies under recent investigation for advanced UC treatment are those targeting DNA synthesis, repair, and gene expression. Taxanes disrupt cell division by inhibiting microtubule function. In addition to the previously discussed 2017 phase III trial that demonstrated the efficacy of docetaxel in combination with ramucirumab (70), as well as the 2018 phase II study that showed improved outcomes in UC patients given docetaxel plus apartosen compared to docetaxel monotherapy (90), paclitaxel has also shown promise as second or third-line combination therapy with cytotoxic chemotherapy and other agents. A 2020 phase II single-arm trial administered paclitaxel, gemcitabine and cisplatin to twenty-one patients with advanced/metastatic UC that progressed after platinum-based chemotherapy. The ORR was 23.8%, and the respective median PFS and OS were 4 and 8.4 months. 71.4% and 42.9% of patients experienced grade 3-4 neutropenia and thrombocytopenia, respectively (91). In the first report that specifically examined paclitaxel with carboplatin therapy for advanced UC patients after platinum-based chemotherapy and pembrolizumab failed, the median PFS and median OS were 9.8 months and 13.0 months, respectively (92). Paclitaxel with ifosfamide and nedaplatin is another potential second-line treatment option. A retrospective study published in 2016 examined 33 patients with metastatic UC who were given this regimen after cisplatin-based therapy failed. The median OS was 10.4 months, the median PFS was 3.5 months and the ORR was 30%. There were no treatment-related deaths, although all patients acquired grade 3-4 neutropenia (93).

The taxane cabazitaxel has been studied as combination therapy with cisplatin in a phase II trial for MIBC patients who are eligible for radical cystectomy. The most common treatment-related AEs were fatigue and nausea and vomiting. Among 26 patients, the ORR was 57.7% (95% CI 36.9%-76.6%). Only 10 patients experienced grade ≥3 AEs (94).

Pemetrexed inhibits multiple folate-dependent enzymes, thereby limiting cancer growth by inhibiting nucleotide synthesis, and has been studied in a handful of trials since 2015. A retrospective analysis also examined the efficacy of pemetrexed as monotherapy in 129 platinum-resistant advanced UC patients. The ORR was 5% and median PFS was 2.4 months (95). Pemetrexed has also been used as combination therapy with various agents. The phase II PECULIAR study involved 42 advanced UC patients, treated with pemetrexed and cisplatin combination therapy; the ORR was 64.3%, median PFS was 6.9 months, and median OS was 14.4 months. 33.33% of the patients experienced grade 3-4 neutropenia (96). Cao et al.’s phase I 2018 trial compared the outcomes of gemcitabine-cisplatin versus pemetrexed-cisplatin in patients with advanced or metastatic UC. The gemcitabine-cisplatin group had response rate of 68% and disease control of 86%, compared to the pemetrexed-cisplatin group’s significantly lower response rate and disease control of 44% and 56%, respectively (97). A phase I trial studying pemetrexed with vinflunine in patients with metastatic UC refractory to platinum chemotherapy suggested that this regimen has poor outcomes. Out of the four patients enrolled in the study, one patient had grade 4 thrombocytopenia, another patient had grade 3 hepatobiliary toxicity, and all patients had progression as defined by RECIST 1.1 criteria (98).

Apatorsen is an antisense oligonucleotide that inhibits heat shock protein 27. In the 2017 phase II Borealis-1 trial, patients with advanced, untreated UC were randomly assigned to receive GC with placebo, 600 mg apatorsen, or with 1000 mg apatorsen. Overall survival was the primary endpoint. There was not a significant survival benefit in patients treated with the combination of GC with 600 mg or 1000 mg apatorsen; however, exploratory analysis showed a trend for improved survival in patients with poor prognostic features (99). The 2018 Borealis-2 trial was a phase II study that examined treatment outcomes when apatorsen was given with the microtubule inhibitor docetaxel in patients with metastatic UC who relapsed after given platinum-based chemotherapy. Patients who received apatorsen with docetaxel had significantly improved OS compared to those who received docetaxel alone, with a median OS of 6.4 versus 5.9 months. In the apatorsen with docetaxel group, common grade 3-5 AEs were leukopenia [27(29%)], neutropenia [33(35%)], anemia [16(17%)], and sepsis [14(15.1%)] (90).

Pralatrexate (PTX) is a new agent that inhibits dihydrofolate reductase (DHFR), and palbociclib isethionate (PAL) is a cell cycle inhibitor evaluated in colon cancer, lung cancer, and HER2 -, ER+ breast cancer. Wang et al.’s retrospective analysis studied the clinical efficacy of PTX with PAL in advanced bladder cancer patients. 42 cases involved PTX + PAL therapy, and 40 cases received GC. In the PTX + PAL group, 33.33% achieved CR and had significantly lower rates of adverse events such as nausea, vomiting and liver function enzyme elevation compared to the GC group. The most common treatment-related AEs in the PTX + PAL group was leukopenia [19(45.2%)] and nausea and vomiting [15(35.7%)] (100).

Another novel agent, fluorocyclopentenyl cytosine (RX-3117), is activated exclusively in the tumor environment and acts by damaging DNA and inhibiting DNA methyltransferase 1, and studies have demonstrated its efficacy for the treatment of pancreatic cancer and advanced UC (117). There is an ongoing phase 2a study involving RX-3117 monotherapy for advanced UC (NCT02030067). The preliminary results suggest that RX-3117 is well-tolerated and able to induce partial response and tumor reduction, with fatigue, diarrhea and nausea and vomiting being the most common AEs (101).

Olaparib inhibits poly ADP ribose polymerase, a DNA repair enzyme, restricting DNA replication in tumor cells. Olaparib is being studied in a phase II trial as combination therapy with durvalumab for patients with unresectable stage IV UC who are ineligible for platinum therapy, and the outcomes will be compared to treatment with durvalumab monotherapy (NCT03459846). Another ongoing phase II trial is studying the effects of neoadjuvant olaparib with durvalumab in patients with resectable stage IV UC prior to surgery (NCT03534492). The preliminary data showed a pathologic CRR of 44.5% with only 8.3% of patients experiencing a grade 3 or 4 AE. Common AEs from this combination therapy include grade 1 nausea and vomiting and anemia (102).

5-fluoro-2’-deoxcytidine (FdCyd) alters gene expression in cancer cells, and tetrahydrouridine (THU) prevents FdCyd breakdown. The safety and antitumor effects of FdCyd with THU in patients with unresectable or metastatic malignancies such as UC that progressed after standard therapy are currently being studied in a phase II trial (NCT00978250).

#### T-Cell Therapies

There is a phase I study investigating chimeric antigen receptor T-cell (CAR-T) therapy for refractory or recurrent stage IV solid malignancies such as bladder cancer. CAR-T therapy utilizes genetic engineering to create specific T-cell receptors that will enable the host immune response to recognize tumor cells. Patients who have a biopsy positive for receptor tyrosine kinase-like orphan receptor 2 will receive this treatment (NCT03960060). CAR-T has proven itself to be promising therapy for B cell malignancies, but its efficacy as treatment for solid tumors has yet to be demonstrated. This is due to the significant challenges to immune function presented by the solid tumor setting, such as limited capacity for lymphocyte invasion, which are not found in the environment of hematologic malignancies (118).

Another ongoing phase I study compares the effects of PF-04518600, an agonist of the memory T-cell protein OX40 (CD134), as monotherapy versus combination therapy with utomilumab (PF-05082566), an agonist of the 4-1BB (CD-137) receptor on T cells and natural killer cells, in patients with advanced or metastatic malignancies including UC (NCT02315066).

An ongoing phase I trial is examining the safety profile of TCR affinity enhancing specific T cell therapy (TAEST16001) for advanced solid tumors such as bladder cancer that failed multi-line treatment. To be eligible for enrollment, patients must have cells that are HLA-A*0201 positive and at least 25% of cells must be NY-ESO-1 positive (NCT03159585).

There is also a phase II trial studying cytokine induced killer cells for management of bladder neoplasms after chemotherapy treatment; this study will enroll about 1500 patients with stage I-III bladder cancer (NCT02489890).

#### Personal Peptide and Virus Vaccinations

There is rapidly growing literature on utilizing cancer peptide vaccinations (PPV) for management of bladder cancer. PPV customizes therapy for a specific individual by accounting for a patient’s HLA markers and host immunity prior to vaccination. The antigens presented by peptide vaccines can trigger specific T-cell responses against tumor activity. S-288310 contains two distinct kinds of peptide vaccines, one derived from M phase phosphoprotein 1 (MPHOSPH1) and one derived from DEP domain containing 1 (DEPDC1), and was shown in a phase I/II study to be safe and effectively induced cytotoxic T-lymphocytes in advanced UC patients. The ORR was 6.3% in 32 patients. After one, two, and three or more regimens, the median OS rates were 14.4, 9.1 and 3.7 months respectively (103). Another phase II trial compared PPV monotherapy to PPV plus salvage chemotherapy in metastatic/advanced upper tract UC patients who had failed platinum-based chemotherapy. The combination therapy group’s median survival time was 13.0 months and there were no severe adverse treatment-related events (104). A phase II randomized trial compared the outcomes of PPV with best supportive care (BSC) versus PPV alone in UC patients who progressed after platinum-based therapy. There was no significant improvement in PFS, but the median OS was 7.9 months in the PPV with BSC group compared to 4.1 months in the BSC alone group, and PPV therapy was well-tolerated. The most common AE was dermatologic reactions at the sites of injection, abdominal pain, peripheral edema, and anemia, but no immune-mediated severe AEs were observed (105).

NEO-PV-01 is a new type of PPV that is specifically designed based on a particular individual’s tumor DNA and can administer up to twenty customized peptides in each dose. It is administered with the adjuvant Poly-ICLC, which helps to stimulate the immune response. An ongoing phase I trial is studying the effects of NEO-PV-01 plus Poly-ICLC in combination with nivolumab for patients with advanced/metastatic melanoma, NSCLC, or UC (NCT02897765).

The modified vaccinia Ankara virus, an attenuated poxvirus vaccine, can potentially improve the host immune response to UC antigens. It is currently being studied in a phase I trial as combination therapy with pembrolizumab in patients with refractory metastatic malignancies including UC that are overexpressing p53 (NCT02432963).

#### Oncolytic Virus Therapy

The oncolytic virus CAVATAK is being studied in another phase I trial as combination therapy with pembrolizumab for patients with either advanced/metastatic NSCLC, melanoma, prostate cancer or UC (NCT02043665).

#### Agents Altering Tumor Microenvironment

Another pathway that is being investigated is the IDO1 mediated immune mechanism. IDO1 is a cytosolic enzyme that changes the tumor microenvironment so that there are greater amounts of kynurenine and lower tryptophan levels; this promotes the activity of T regulator cells and inhibits effector T cells. Navoximod is a small molecule that inhibits IDO1. Jung et al.’s phase Ib trial examined the safety of combination therapy with navoximod and atezolizumab in advanced/metastatic solid tumors including urothelial carcinoma. 158 patients were enrolled, and the study involved a dose escalation and an expansion stage. Adverse effects reporting period continued until 60 days after the final dose of navoximod and/or atezolizumab. 22% of patients experienced treatment related adverse effects of grade 3 or higher, the most common being rash [14(9%)] (106).

NCB024360 (epacadostat) is another IDO1 inhibitor whose safety and efficacy have been recently studied in a phase I/II when given in combination with pembrolizumab for malignancies including advanced bladder cancer. For the highest dose of INCB024360 given in phase I (300 mg BID), 8 of 20 participants experienced serious adverse effects. In phase II, patients were given 100 mg BID and the all-cause mortality rate was 53.14%. The most common grade ≥3 treatment-related AE was rash (NCT02178722).

Arginase depletes the tumor environment of arginine, leading to suppressed function of the immune cells. Thus, arginase inhibition can restore the host immune defense against tumor cells. The arginase inhibitor INCB001158 is currently being studied in a phase I/II trial for patients with advanced/metastatic malignancies such as UC. Patient outcomes from INCB001158 monotherapy will be compared to the outcomes produced by INCB001158 combined with pembrolizumab (NCT02903914).

### Future Perspectives for NMIBC

#### DNA Synthesis, Replication

The anthracycline agent pirarubicin has been studied as combination treatment with hyaluronic acid following TURBT. Huang et al.’s 2015 trial has suggested that this combination therapy is safe and more tolerable than pirarubicin alone in NMIBC patients after TURBT. There was no statistically significant difference in recurrence between the combination therapy group and the pirarubicin only group (18.8% *vs*. 25.4%, P = 0.27). The most common treatment-related AEs in both groups were odynuria, cystitis and anxiety, but no severe AEs occurred in either arm (107).

Cabazitaxel in combination with gemcitabine and cisplatin administered as intravesical therapy has also shown in a phase I study to be a well-tolerated regimen in patients with NMIBC that failed intravesical BCG therapy. The initial partial rate was 94%, and the ORR was 89%. The 1-year recurrence free survival was 0.83, with a 2-year estimated RFS of 0.64. There were no dose-limiting toxicities (108).

Apaziquone (Qapzola) is an analog of mitomycin C, a crosslinking agent that inhibits DNA synthesis. It is being studied as adjuvant therapy in NMIBC patients; two phase III trials are currently examining the efficacy of apaziquone in NMIBC patients after TURBT. One trial compares an 8 mg apaziquone treatment group to a placebo group (NCT03224182), and the other compares 4 mg apaziquone versus 8 mg apaziquone treatment groups relative to a placebo group (NCT02563561).

Vicinium contains a recombinant fusion protein called that VB4-845, which binds to the epithelial cell adhesion molecule antigen that is on the surface of cancer cells. Once internalized, this protein can block protein synthesis, thereby inducing cell death. A Phase III trial is currently studying the efficacy of Vicinium for NMIBC *in situ* or high-grade invasive papillary bladder cancer who have failed BCG therapy (NCT02449239).

Dendritic cell-based immunotherapy has also been proposed as a feasible regimen in advanced UC patients. Dendritic cells (DC) are a type of antigen presenting cells that can trigger both the innate and adaptive immune response. Ogasawara et al.’s phase I/II trial examined vaccination therapy involving Wilms Tumor 1 peptide-pulsed dendritic cells and OK-432 in combination with molecular targeted therapy or standard chemotherapy. Five renal cell carcinoma and five NMIBC patients were enrolled. The dendritic cells and OK-432 were given every two weeks, with seven doses total. Salvage chemotherapy was provided as adjuvant treatment in UC patients. None of the patients had adverse events from the leukapheresis period. Other than grade 3 hematological adverse effects that occurred in 5 patients, all patients’ treatment related adverse effects were grade 1 or 2. The two patients with metastatic UC died following DC vaccination, but three of the NMIBC patients achieved stable disease. Only 4 patients experienced grade 3 AEs, all of which were hematologic (leukocytopenia, neutropenia, anemia) (109).

#### Oncolytic Viruses

A phase I trial studied CAVATAK, a coxsackievirus agent that targets ICAM-1 in NMIBC patients scheduled for TURBT. By binding to adhesion molecules like ICAM-1, the virus can enter and subsequently lyse tumor cells. A phase I trial administered CAVATAK to NMIBC patients; 9 patients received CAVATAK alone and 6 received it after first being treated with mitomycin C. The safety profile was manageable, with only 1 patient who received CAVATAK alone experiencing a serious adverse effect. Tissue biopsies also demonstrated that CAVATAK upregulated immune checkpoint inhibitory genes such as PD-L1 and LAG3 and chemokines associated with Th1. Common AEs included hematuria and dysuria (110).

Enadenotucirev is a chimeric adenovirus that carries out oncolytic functions by creating a pro-inflammatory environment and inducing non-apoptotic tumor cell death. A phase I mechanism of action study enrolled patients with histologically confirmed early stage renal cell cancer, colorectal cancer, non-small cell lung cancer, or UC, with a tumor size of at least 3 cm diameter who are scheduled for resection and treated them with one cycle of enadenotucirev. In tumor samples obtained after treatment and resection surgery, delivery of enadenotucirev was detected in most tumor samples and produced an immune response by evidence of high local CD8 cell infiltration in 80% of tested tumor cells, with little to no activity observed in regular tissue. Treatment-related AEs included asthenia, chills, neutropenia and pyrexia, but no grade 3-4 AEs occured (111).

CG0070 is another oncolytic adenovirus, which specifically targets UC cells. Its mechanism of action involves triggering the lysis of cancer cells and releasing various antigens that lead to a widespread anti-tumor host response. The interim findings of a phase II study that administered CG0070 to NMIBC patients refractory to intravesicular BCG therapy and refused cystectomy (NCT02365818) showed that the 6-month CR among all 45 patients was 47%, with a manageable toxicity level. The most common treatment-related AEs were bladder spasms, hematuria, dysuria and urgency. Grade III treatment-related AEs were dysuria (3%) and hypotension (1.5%), but no grade IV/V AEs occurred (119).

#### Virus Vector Vaccine

PANVAC is a virus vector that contains the CEA and MUC-1 genes, which are commonly found in solid malignancies, and costimulatory molecules to improve host T-cell response. A phase I trial compared PANVAC alone with PANVAC plus BCG therapy for patients with high-grade NMIBC who had previously failed at least one course of intravesicular BCG therapy. The 6-month RFS was 66% in the PANVAC plus BCG group, compared to 60% in the PANVAC alone group. In the combination therapy group, the all-cause mortality was 20% (3 patients out of 15), versus 13.33% in the PANVAC monotherapy group (2 of 15). Serious AEs in the combination therapy arm include postoperative hemorrhage, hematuria, urinary retention, and urinary tract obstruction (NCT02015104).

#### Personal Peptide Vaccination

Peptide vaccines have also been studied as therapy for NMIBC. In non-muscle invasive UC, a phase II trial researched both MPHOSPH1 and DEPDC1 for prevention of NMIBC recurrence after undergoing TURBT. Recurrence free survival (RFS) was 74%, and was longer in patients who had a positive reaction to at least one peptide vaccine in combination with the BCG vaccine (p = 0.0019). Out of 132 patients, only 2 experienced a grade 3 event. The most common grade 1-2 AEs were urinary frequency, hematuria, and urinary tract pain (112).

#### MAGE-A3 Vaccine

The results of a phase I trial suggest that the melanoma-associated antigen (MAGE-A3) tumor vaccine, when combined with BCG is a safe regimen for NMIBC that does not disrupt the function of BCG. Following intravesical BCG and vaccine administration, the most common AEs were local injection site tenderness, urological symptoms and systemic symptoms, but BCG treatment did not exacerbate these AEs (113).

#### Targeting FGFR

There is an ongoing study involving 4 patients with NMIBC treated with the pan-FGFR inhibitor infigratinib, and cystoscopy and cytology will be used to assess tumor response (NCT02657486).

#### Targeting the mTOR Pathway

Rapamycin, also known as sirolimus, is a macrolide agent that inhibits both the mTORC1 and mTORC2 complexes and thus limits cancer proliferation through multiple mechanisms (120). There is an ongoing combined phase I/II trial that examines role of albumin-bound rapamycin nanoparticles in patients with recurrent NMIBC or muscle-invasive bladder cancer that is refractory to BCG therapy (NCT02009332). There is also a phase II trial in the recruitment process investigating encapsulated rapamycin as a secondary prevention agent for NMIBC (NCT04375813).

#### Angiogenesis

APL-1202 is a methionine aminopeptidase type II inhibitor, limiting angiogenesis by inhibiting the proliferation of endothelial cells. It is being currently being studied in a phase Ib trial in NMIBC patients resistant to one induction course of BCG. The safety of APL-1202 monotherapy will be assessed and compared to the outcomes of patients that received APL-1202 with concurrent BCG (NCT03672240).

#### Nadofaragene Firadenovec

The gene transfer vector nadofaragene firadenovec has been studied as therapy for not only advanced/metastatic UC, but NMIBC as well. A phase II study involving patients with relapsed or BCG refractory NMIBC were given intravesical nadofaragene firadenovec. Out of 40 patients, 14 remained recurrence-free for 12 months after receiving treatment, and the therapy was well-tolerated. The most common treatment-related AEs were micturition urgency (16[40%]), dysuria (16[40%]), and fatigue (13[32.5%]) (114). There is an ongoing phase III study evaluating this agent in patients with high grade NMIBC refractory to intravesicular BCG therapy, and CR rate will be assessed (NCT02773849).

#### Immunomodulators

Imiquimod is a toll-like receptor 7 agonist that regulates various functions including cytokine release, angiogenesis, and immune cell proliferation. Although it is typically used as topical treatment for skin malignancies, and has a liquid formulation called TMX-101 (Vesimune) that has been safely used in a phase I trial involving non-muscle invasive bladder cancer patients (115). A phase II trial involving 12 UC *in situ* patients were treated with 6 weekly intravesical doses of TMX-101, and half of these patients received at least 2 induction courses of BCG. Only 1 patient experienced a treatment related adverse effect greater than grade 2 (urinary tract infection), and 2 patients had a clinical response. Additionally, there were significant elevations in urinary levels of various interleukins and VEGF after treatment (116).

## Conclusion

Pioneering research in the management of bladder cancer over the past decade has introduced a surge of new therapeutic agents with various novel molecular targets through rapidly advancing technology. These therapies have revolutionized the field of urothelial cancer and provide feasible alternatives to traditional platinum-based chemotherapy regimens. Many of these agents are still in the early phases of investigation to examine safety and efficacy. Although a handful of agents have received FDA approval and have shown significant promise, more research is required to identify additional targets, fully elucidate their molecular mechanisms, and determine their long-term outcomes.

## Author Contributions

BN and AH: Conceptualization, Methodology, Data curation, Original draft preparation & Illustration. BJ: Conceptualization, Reviewing and Editing. All authors contributed to the article and approved the submitted version.

## Conflict of Interest

The authors declare that the research was conducted in the absence of any commercial or financial relationships that could be construed as a potential conflict of interest.

## Publisher’s Note

All claims expressed in this article are solely those of the authors and do not necessarily represent those of their affiliated organizations, or those of the publisher, the editors and the reviewers. Any product that may be evaluated in this article, or claim that may be made by its manufacturer, is not guaranteed or endorsed by the publisher.
